# *Gynostemma Pentaphyllum* ameliorates CCl_4_-induced liver injury via PDK1/Bcl-2 pathway with comprehensive analysis of network pharmacology and transcriptomics

**DOI:** 10.1186/s13020-024-00942-w

**Published:** 2024-05-15

**Authors:** Linlan Hu, Xin Zhao, Xian He, Yafei Guo, Hanxiao Cheng, Shaoting Chen, Guangde Zhou, Jiabo Wang, Yawen Lu

**Affiliations:** 1 College of Pharmacy, Henan University of Chinese Medicine, Zhengzhou, 450046 China; 2 School of Traditional Chinese Medicine, Capital Medical University, No.10 Outside You’anmen, Fengtai District, Beijing, 100069 China; 3 Centre for Clinical Pathology, Beijing You’an Hospital, Capital Medical University, No.8 Outside You’anmen, Fengtai District, Beijing, 100069 China; 4 State Key Laboratory for Quality Ensurance and Sustainable Use of Dao-DiHerbs, Beijing, 100700 China

**Keywords:** *Gynostemma pentaphyllum*, Gypenoside A, Liver protection, Hepatocyte apoptosis, Phosphoinositide dependent protein kinase-1 (PDK1)

## Abstract

**Background:**

*Gynostemma pentaphyllum* (Thunb.) Makino, commonly known as “southern ginseng”, contains high amounts of ginsenoside derivatives and exhibits similar biological activities with *Panax ginseng* (C. A. MEY) (ginseng), which is usually used as a low-cost alternative to ginseng. *G. pentaphyllum* has therapeutic effects on liver diseases. However, the mechanisms underlying its hepatoprotective action have not been fully elucidated.

**Methods:**

The protective effects of the ethanolic extract of *G. pentaphyllum* (GPE) were evaluated using an experimental carbon tetrachloride (CCl_4_)-induced liver disease model. Potential targets of GPE were predicted using the “Drug-Disease” bioinformatic analysis. Furthermore, comprehensive network pharmacology and transcriptomic approaches were employed to investigate the underlying mechanisms of GPE in the treatment of liver disease.

**Results:**

The pathological examinations showed that GPE significantly alleviated hepatocyte necrosis and liver injury. GPE significantly downregulated Bax and cleaved-PARP expression and upregulated Bcl-2 expression during CCl_4_-induced hepatocyte apoptosis. We compared the effects of four typical compounds in GPE -a ginsenoside (Rb3) shared by both GPE and ginseng and three unique gypenosides in GPE. Notably, Gypenoside A (GPA), a unique saponin in GPE, markedly reduced hepatocyte apoptosis. In contrast, ginsenoside Rb3 had a weaker effect. Network pharmacology and transcriptomic analyses suggested that this anti-apoptotic effect was achieved by upregulating the PI3K/Akt signaling pathway mediated by PDK1.

**Conclusions:**

These results suggested that *G. pentaphyllum* had a promising hepatoprotective effect, with its mechanism primarily involving the upregulation of the PDK1/Bcl-2 signaling pathway by GPA, thereby preventing cell apoptosis.

**Graphic Abstract:**

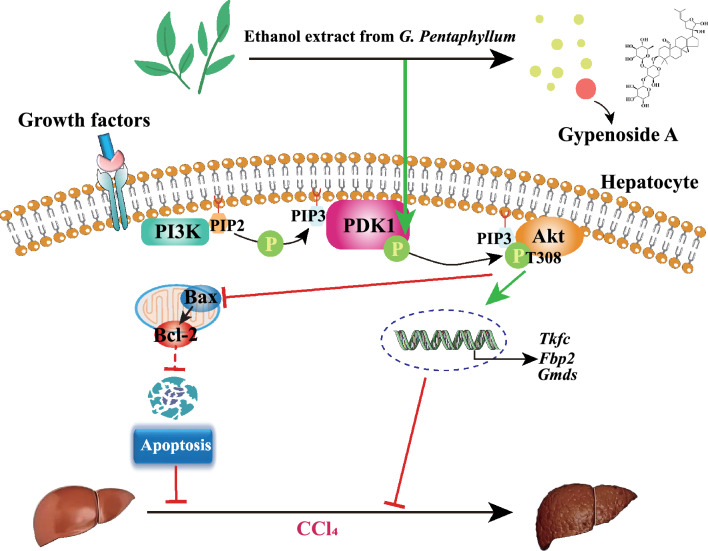

**Supplementary Information:**

The online version contains supplementary material available at 10.1186/s13020-024-00942-w.

## Introduction

*Gynostemma pentaphyllum* (Thunb.) Makino has a long history of use as a traditional medicine and dietary supplements in China. In 2002, the Chinese Ministry of Public Health of China recognized *G. pentaphyllum* as a functional food. Saponins are the primary bioactive components of G. pentaphyllum. They play a significant role in its diverse biological activities and clinical effects. *G. pentaphyllum* is a unique plant outside the family Araliaceae that contains ginseng saponins and at least eight ginsenosides identical to the saponins found in *Panax ginseng* (C. A. MEY) (ginseng). These shared compounds provide *G. pentaphyllum* with a tonic function similar to ginseng and for this reason, *G. pentaphyllum* is praised as “Southern ginseng” and widely used as a low-cost alternative to ginseng [1]. *G. pentaphyllum* has excellent research and development value.

Liver injury is a critical stage in the progression from chronic liver disease to cirrhosis and poses a serious threat to patient health [2]. The occurrence and development of liver injury are complex processes involving both liver parenchymal cells and non-parenchymal cells, including sinusoidal endothelial cells, hepatic stellate cells (HSCs), Kupffer cells, and different types of lymphocytes. Among them, HSCs are the primary source of collagen synthesis and secretion in the liver, and are commonly considered as effector cells of liver fibrosis. Kupffer cells are special macrophages participating in HSC activation and regulation alongside other lymphocyte types. Excessive and persistent hepatocyte apoptosis is a crucial hallmark of liver injury progression and serves as a trigger for liver injury and fibrosis [3, 4]. Therefore, targeting hepatocyte apoptosis is a critical approach for treating liver injury. Treatments and drugs that focus on targeting liver parenchymal cell damage while considering other hepatic cells are required to treat clinical liver diseases.

The phosphatidylinositol-3 kinase/protein kinase B (PI3K/Akt) pathway, is a key survival pathway that targets multiple molecules involved in anti-apoptosis, antioxidant defense, and protein synthesis [5]. Phosphoinositide dependent protein kinase-1 (PDK1), a serine/threonine kinase of the Protein Kinase A, G, and C family, is a fundamental component of the PI3K/PDK1/Akt pathway [6]. However, PDK1 expression and function in liver injury remains unexplored. Further exploration of PDK1 expression and function and its relationship with liver injury may provide new targets for treating liver diseases.

In vivo and in vitro studies have indicated that the ethanol extract of *G. pentaphyllum* (GPE) exhibits a range of favorable bioactivities, including antioxidant, anti-inflammatory, and antilipidemic effects [7], suggesting that GPE can regulate hepatic cellular homeostasis. This study investigated the protective effects of GPE in mice with carbon tetrachloride (CCl_4_)-induced liver injury. Network pharmacology and transcriptomics analyses revealed that GPE and Gypenoside A (GPA) upregulated the expression of proteins involved in the PI3K/PDK1/Akt signaling pathway, alleviating CCl_4_-induced liver injury and hepatocyte apoptosis. Therefore, *G. pentaphyllum* is expected to be a safe dietary supplement and medicinal material for treating liver injury.

## Materials and methods

### Chemicals and reagents

*G. pentaphyllum* leaves (Cat: 20230615) were acquired from Beijing Bencao Fangyuan Co., Ltd. (Beijing, China). CCl_4_ was obtained from Boer’s (Shanghai, China). Olive oil was purchased from Yuanye Biotechnology Co., Ltd. (Shanghai, China). Ginsenoside Rb3 (Cat: S9208) and Gypenoside XLIX (Cat: S9177) were purchased from Selleck (Shanghai, China). Gypenoside A (Cat: HY-N2440), Gypenoside XLVI (Cat: HY-N6252) and GSK2334470 (Cat: HY-14981) were purchased from MedChem Express (Shanghai, China). The aspartate transaminase (AST, Cat: BC1565), alanine transami-nase (ALT, Cat: BC1555) and hydroxyproline (HYP, Cat: BC0255) were purchased from Solarbio Life Sciences (Beijing, China).

### Preparation of GPE

100 g leaves and 1 liter of 80% ethanol aqueous solution were mixed thoroughly and sonicated for 1 h, gfiltered, and the filtrate collected and stored in the refrigerator at 4 ℃. Next, the residue was added to 1L of 80% aqueous ethanol aqueous solution, and the procedure was repeated to collect the filtrate. The filtrates from the two filtration and vacuum drying rounds were combined to obtain crude GPE powder.

### Animals

We obtained 30 male C57BL/6 J mice (16–18 g) from Beijing Vital River Laboratory Animal Technology Co., Ltd. (SCXK (JING) 2021–0011). The mice were kept under controlled environmental conditions, with a temperature of 25 ± 2 °C and a relative humidity of 50 ± 10%. A 12 h light/dark cycle was established, and all mice were provided water and standard chow throughout the study period. The mice were intraperitoneally with 5% CCl_4_ or an equivalent amount of olive oil daily for 6 weeks. The mice were injected once daily for the first 2 weeks and once every 2 d for the next 4 weeks. GPE was administered by gavage at a dose of 500 mg/kg/day from the beginning of the study for 6 weeks. An equal volume of 0.5% sodium carboxymethyl cellulose was administered to the control group. The Capital Medical University Animal Ethics Committee approved this study (Permit number: AEEI-2023-132).

### Histopathological examination

Hepatic pathological changes were examined using hematoxylin and eosin (H&E), Masson’s trichrome, and Sirius red staining. Collagen deposition and fibrosis were evaluated using ImagePro Plus 6 software (Media Cybernetics, Inc.). Hepatocyte apoptosis was assessed by terminal deoxynucleotidyl transferase-mediated dUTP Nick-End Labeling (TUNEL) staining (Servicebio, Wuhan, China).

### Network pharmacology and bioinformatics-based analysis

*G. pentaphyllum* compounds were sourced from the TCMSP database (http://lsp.nwsuaf.edu.cn/tcmsp.php) and screened based on criteria of oral bioavailability critrria ≥ 30% and drug-likeness ≥ 0.18. *G. pentaphyllum* potential target proteins were predicted using SwissTargetPrediction (http://www.swisstargetprediction.ch/) Using the Keyword “Liver Injury” we identified liver injury-related targets from the Online Mendelian Inheritance in Man database (http://www.omim.org/) and Genecards (http://www.genecards.org), a comprehensive database that provides information on genes, their products, and biomedical applications.

We constructed an interaction network for *G. pentaphyllum* active components and putative targets in treating liver injury using their interaction data. The interaction network was visualized using Cytoscape software (Version 3.9.0). We analyzed the degree, betweenness, and closeness centrality, using the network Analyzer plugin in Cytoscape. Thus, providing insights into the topological significance of nodes within the network. In addition, a protein-protein interaction network was established to explore the interplay between these targets using Cytoscape (version 3.9.0). Kyoto Encyclopedia of Genes and Genomes (KEGG) analysis was conducted using the Database for Annotation, Visualization, and Integrated Discovery (https://david.ncifcrf.gov/). Molecular docking was conducted to screen for proteins exhibiting superior binding affinities for GPA using the SYBYL-X 2.0 software.

### Transcriptomic analysis

Total RNA was isolated from primary mouse hepatocytes using Trizol reagent after treatment with DMSO, GPE, or GPA in the presence of 8 mM CCl_4_ for 24 h. Subsequently, cDNA libraries were generated following the manufacturer’s protocols, including the use of the NEBNext^®^ Ultra^™^ Directional RNA Library Prep Kit, NEBNext^®^ Poly (A) mRNA Magnetic Isolation Module, and NEBNext^®^ Multiplex Oligos (New England Biolabs, Ipswich, MA, USA). Differentially expressed genes (DEGs) were identified based on the criteria of |log_2_FC|> 0.585 and a *p*-value < 0.05, which indicated statistical significance.

### Cell culture-primary hepatocyte isolation

Primary hepatocytes were isolated using the standard 2-step perfusion technique [8]. The liver was perfused with 50 mL of HBSS buffer through the inferior vena cava at a controlled flow rate of 5 mL/min. Subsequently, a 25 mL solution containing 0.5 mg/mL collagenase was perfused at a controlled flow rate of 3 mL/min. Following perfusion, the liver was extracted from the abdominal cavity, and the hepatocytes were liberated into DMEM using sterile surgical scissors. The resulting cell suspension underwent filtration through a 70 μm cell strainer. After centrifugation, hepatocytes were subjected to low-speed gradient centrifugation (50 g, 3 min). The viability of the isolated hepatocytes reached approximately 90%, as determined by Trypan blue staining. Finally, the hepatocytes were seeded into 6-well plates and cultured in DMEM supplemented with 10% FBS and 1% penicillin-streptomycin.

### Measurement of AST, ALT and HYP

AST, ALT, and HYP serum levels of the mice were quantified using commercial kits according to the manufacturer’s protocol (Solarbio Life Sciences). For primary mouse hepatocytes, supernatants were collected post-treatment, and AST and ALT enzymatic activities were determined according to the manufacturer’s protocols (Solarbio Life Sciences).

### Western blotting assay

Liver and hepatocyte proteins were extracted using RIPA buffer supplemented with protease inhibitor and centrifuged at 12,000 g for 15 min. The protein concentration was determined using the BCA method. Subsequently, the protein samples were mixed with a loading buffer, denatured in boiling water for 5 min, and subjected to electrophoretic separation on sodium dodecyl sulfate-polyacrylamide gels. The separated proteins were then transferred to PVDF membranes. Following the transfer, the membranes were incubated overnight at 4 °C with primary antibodies, washed, and then incubated with secondary antibodies (Table S1). Finally, the target protein bands were visualized using a Tanon imaging device and chemiluminescence kit.

### Statistical analysis

All values presented in the figures and text are depicted as mean ± standard error of the mean (SEM). Statistical analyses were conducted using a one-way ANOVA, followed by the Bonferroni post hoc test for multiple comparisons. *P*-value < 0.05 was considered significant. *p < 0.05, **p < 0.0001: versus indicated group.

## Results

### ***GPE improved CCl***_***4***_***-induced liver injury in mice***

**Fig. 1:**
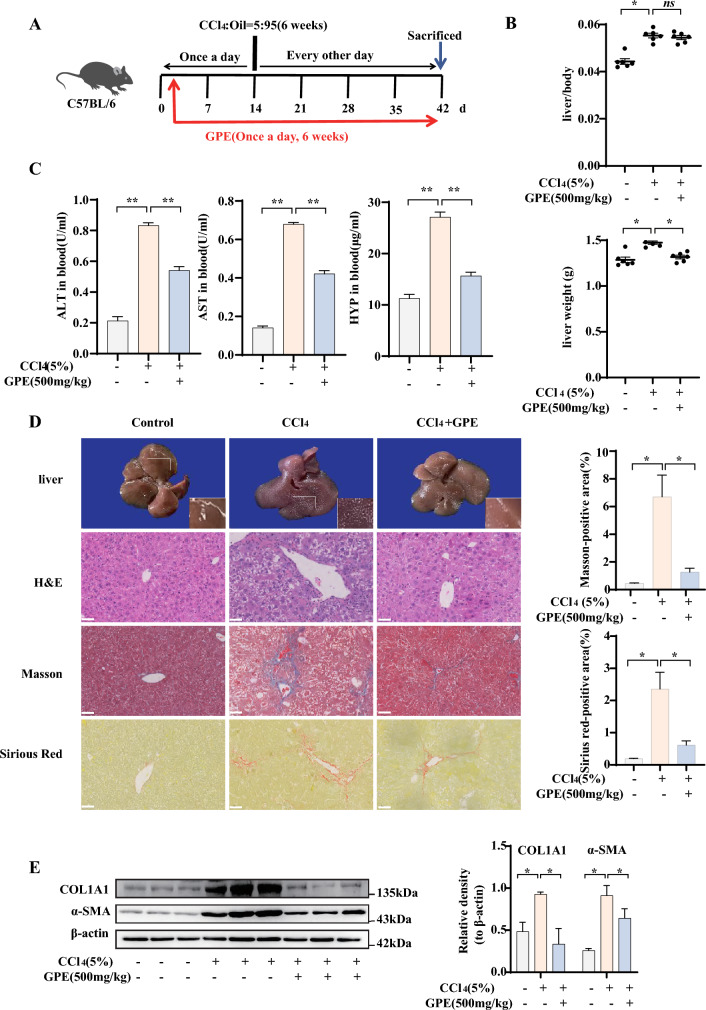
GPE ameliorated CCl_4_-induced liver injury in mice. Mice were injected intraperitoneally with 5% carbon tetrachloride (CCl_4_) or an equivalent amount of olive oil daily for 6 weeks. The mice were injected once daily for the first 2 weeks and then once every 2 d for the next 4 weeks. The ethanol extract of *G. pentaphyllum* (GPE) was administered by gavage at a dose of 500 mg/kg/day from the beginning of modeling for a total of 6 weeks. An equal volume of 0.5% sodium carboxymethylcellulose was administered to the control group. **A** Workflow of mouse experiments. **B** Ratio of liver weight to body weight and liver weight in various groups (n = 6). **C** Serum alanine aminotransferase (ALT), aspartate aminotransferase (AST), and hydroxyproline (HYP) (n = 6). **D** Hematoxylin and eosin (H&E) and Masson and Sirius Red staining of liver tissues (n = 3). Scale bar: 50 μm. (**E**) Protein expression of collagen 1A1(COL1A1) and alpha smooth muscle (α-SMA) in the liver (n = 6). * *p* < 0.05, ** *p* < 0.001: versus indicated group

The CCl_4_-induced chronic liver fibrosis model is a widely accepted experimental model for studying liver injury and fibrosis [9]. In the organism, CCl_4_ initiates lipid peroxidation through hepatic activation, resulting in hepatocyte degeneration, necrosis and liver fibrosis formation [10]. To confirm the GPE curative effect on liver injury, we first observed its effects in a long-term low-dose CCl_4_-induced liver fibrosis model mice (Fig. 1A). Despite having no significant influence on ratio of liver weight to body weight, GPE reduced the liver weight gained in CCl_4_-induced mice (Fig. 1B). Long-term CCl_4_ use resulted in severe liver injury. In contrast, oral administration of GPE reduced the elevated levels of serum AST and ALT (Fig. 1C). Compared with the control group, GPE reversed the CCl_4_-induced impairment of liver surface smoothness (Fig. 1D). H&E staining showed that GPE reduced inflammatory cell infiltration and relieved hepatic cell edema. Masson’s and Sirius red staining further confirmed that GPE reduced hepatic collagen deposition. Consistently, GPE administration reduced HYP levels in circulating blood (Fig. 1C). Besides, we examined the levels of collagen 1A1(COL1A1) and alpha smooth muscle (α-SMA) in the liver and the protein levels of COL1A1 and α-SMA were significantly decreased in the GPE treatment group (Fig. 1E). These results demonstrated that GPE plays a protective role against liver injury and fibrosis.

### Network pharmacology predicted potential signaling pathways in the inhibitory effect of GPE on liver injury

**Fig. 2:**
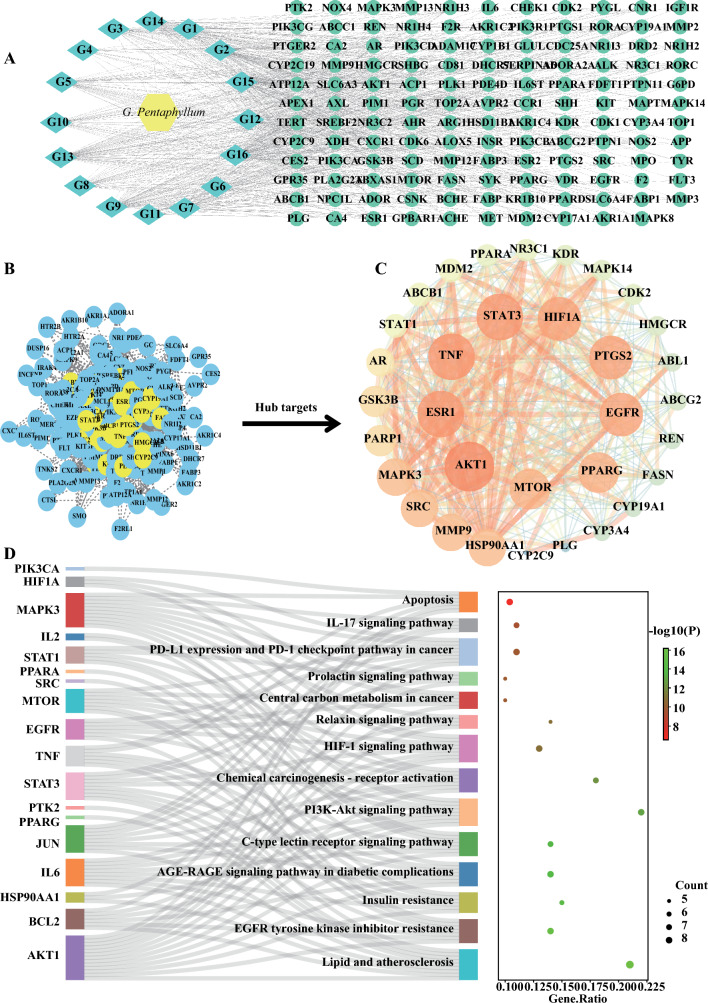
Network pharmacology predicted potential signaling pathways in *G. pentaphyllum* inhibitory effect on liver injury. *G. pentaphyllum* targets in liver injury was predicted using network pharmacological analysis. **A** The compound-target network was established using Cytoscape 3.9.0 software. The yellow hexagon represents *G. pentaphyllum*, the olive-green circles represent the bioactive compounds of *G. pentaphyllum*, and the bottle-green diamonds represent *G. pentaphyllum*-regulated liver injury targets. **B**, **C** Hub targets were identified from the protein-protein interaction (PPI) network using Cytoscape 3.9.0 software. **D** Kyoto Encyclopedia of Genes and Genomes (KEGG) enrichment analysis of 177 *G. pentaphyllum*-regulated liver injury targets using Metascape

Next, a network pharmacology analysis was conducted to understand the mechanism by which GPE inhibits CCl_4_-induced liver injury (Fig. S1A). Initially, 296 potential targets of *G. pentaphyllum* and 3416 potential targets of liver injury were identified using online databases. From this, 177 overlapping targets were selected as *G. pentaphyllum*-regulated liver injury targets (Fig. S1B), and 16 compounds associated with these targets were deemed effective (Table S2). Subsequently, a compound-target network was established, and related functional enrichment analysis was performed (Fig. 2A). KEGG enrichment analysis indicated a close association of these targets with various metabolic signaling pathways, especially the apoptosis signaling pathway (Fig. 2D). Furthermore, a PPI network of the 177 targets was constructed using the Cytoscape software, revealing 33 hub targets (Fig. 2B, C), with AKT1 exhibiting the highest degree value. These findings suggested that GPE therapeutic effect on liver fibrosis might be mediated by regulating apoptotic signaling pathways and AKT1 plays an essential role in this process.

### GPE inhibited CCl_4_-induced hepatocyte apoptosis in vivo

**Fig. 3:**
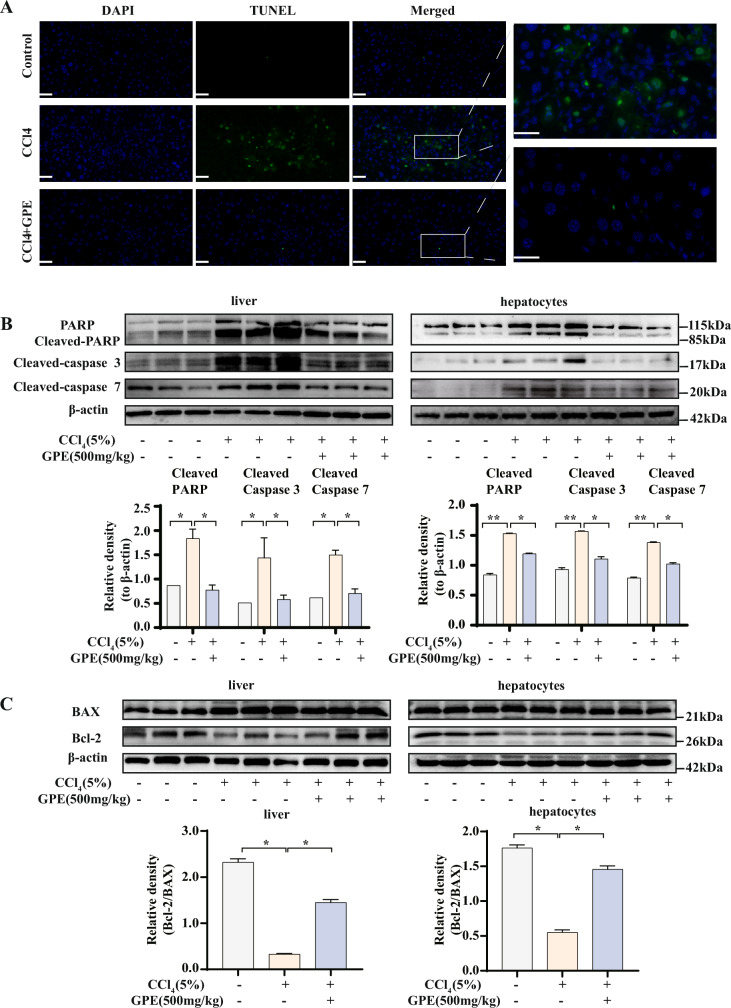
GPE inhibited CCl_4_-induced hepatocyte apoptosis. Mice were intraperitoneally injected with 5% carbon tetrachloride (CCl_4_) or an equivalent amount of olive oil daily for 6 weeks. The mice were injected once daily for the first 2 weeks and then once every 2 d for the next 4 weeks. The ethanol extract of *G. pentaphyllum* (GPE) was administered by gavage at a dose of 500 mg/kg/day from the beginning of modeling for a total of 6 weeks. An equal volume of 0.5% sodium carboxymethyl cellulose was administered to the control group. **A** Terminal deoxynucleotidyl transferase dUTP nick-end labeling (TUNEL) staining of the liver tissue (n = 3). Scale bar: 50 μm. **B** Protein expression levels of Cleaved-PARP, Cleaved-caspase 3, and cleaved-caspase 7 in the livers and hepatocytes isolated from the livers (n = 6). **C** Protein expression levels of Bcl-2/Bax in the liver and hepatocytes isolated from the liver (n = 6). * *p* < 0.05, ** *p* < 0.001: versus indicated group

Hepatocyte apoptosis is involved in the occurrence and development of various of acute and chronic liver diseases. It is an essential mechanism of hepatocyte injury [11]. This study investigated whether GPE is beneficial against liver injury by inhibiting hepatocyte apoptosis. We investigated GPE anti-apoptosis activity in mouse hepatocytes using TUNEL staining. The result showed that a large number of TUNEL-positive cells appeared in the CCl_4_-induced group, indicating that apoptosis occurred. GPE reversed the increase in TUNEL-positive cells induced by CCl_4_ and reduced hepatocyte apoptosis (Fig. 3A). Cleaved-PARP, cleaved caspase-3, and cleaved caspase-7 are typical features of apoptosis. Western blotting showed that the protein expression of Cleaved-PARP, Cleaved-caspase 3, and Cleaved-caspase 7 increased in the CCl_4_-induced group and was decreased when GPE treatment was administered simultaneously (Fig. 3B), further verifying GPE beneficial actions on hepatocyte apoptosis. Bcl-2 and Bax protein are two essential proteins that regulate apoptosis, and the reduced the Bcl-2/ Bax ratio strongly confirmed the role of GPE in hepatocyte apoptosis (Fig. 3C). These results demonstrated that GPE inhibits CCl_4_-induced hepatocyte apoptosis in vivo.

### GPE and GPA prevented CCl_4_-induced hepatocyte apoptosis in vitro

**Fig. 4:**
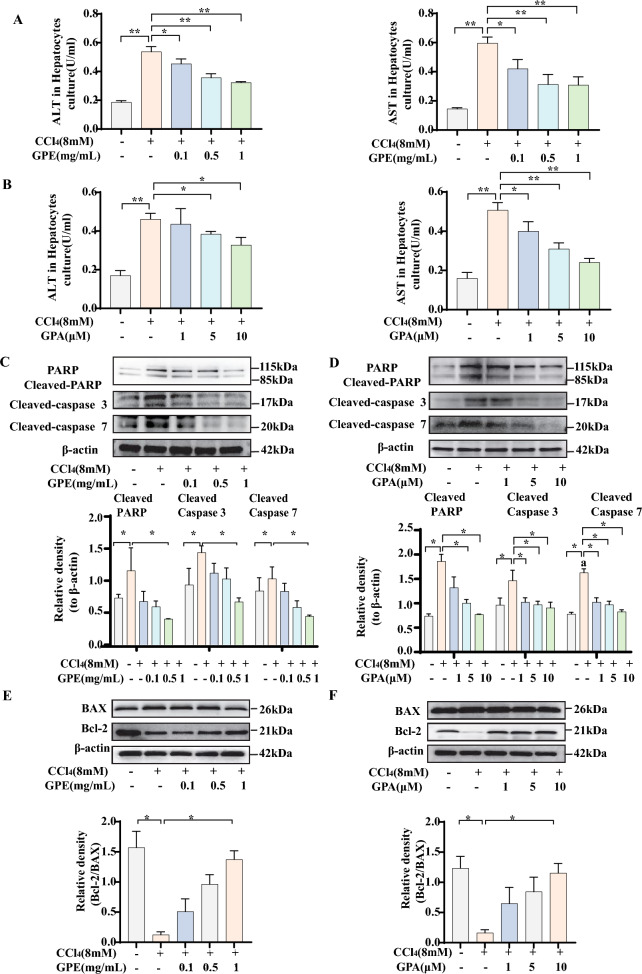
GPE and Gypenoside A (GPA) prevented CCl_4_-induced hepatocyte apoptosis in vitro. Primary mouse hepatocytes were treated with DMSO or the ethanol extract of *G. pentaphyllum* (GPE) or Gypenoside A (GPA) in the presence of 8 mM carbon tetrachloride (CCl_4_) for 24 h. **A**, **B** The levels of alanine aminotransferase (ALT) and aspartate aminotransferase (AST) in the hepatocytes culture-medium (n = 6). **C**, **D** The protein expression levels of Bcl-2/Bax in the hepatocytes (n = 6). **E**, **F** The protein expression levels of PARP/Cleaved-PARP, Cleaved-caspase 3 and Cleaved-caspase 7 in the hepatocytes (n = 6). * *p* < 0.05, ** *p* < 0.001: versus indicated group

We studied the GPE anti-apoptotic effect and the main components of GPE anti-apoptotic mouse hepatocytes. GPA, GP-XLIX and GP-XLVI were the main triterpenoids isolated from GPE, whereas Rb3 is one of the dominant active components in *G. pentaphyllum* and ginseng [12]*.* Fig S2A shows the structures of these compounds. We investigated the anti-apoptotic effects of GPE, GPA, GP-XLIX, GP-XLVI, and Rb3 in a CCl_4_-induced hepatocyte apoptosis model. ALT and AST levels in the cell culture medium showed that GPE and GPA alleviated hepatocyte damage dose-dependently when exposed to CCl_4_ (Fig. 4A, B). The reduced expression of cleaved-PARP, cleaved caspase-3, and cleaved caspase-7 further confirmed the anti-apoptotic effects of GPE and GPA (Fig. 4C, D). GPE and GPA consistently decreased the Bcl-2/Bax ratio in CCl_4_-induced hepatocytes (Fig. 4E, F). However, GP-XLIX and GP-XLVI had no significant effect on the anti-apoptotic phenotype (Fig. S2A). Rb3 showed a tendency to alleviate apoptosis, but its effects were weaker than those of GPA (Fig. S2B). These results indicated that GPE and its unique active component, GPA, exhibits remarkable hepatoprotective and anti-apoptotic effects in vitro. And GPA might as well be the main active component contributing to the anti-apoptotic effect of GPE.

### Transcriptomics analysis revealed the signaling pathways involved in the inhibitory effect of GPE and GPA on liver injury

**Fig. 5:**
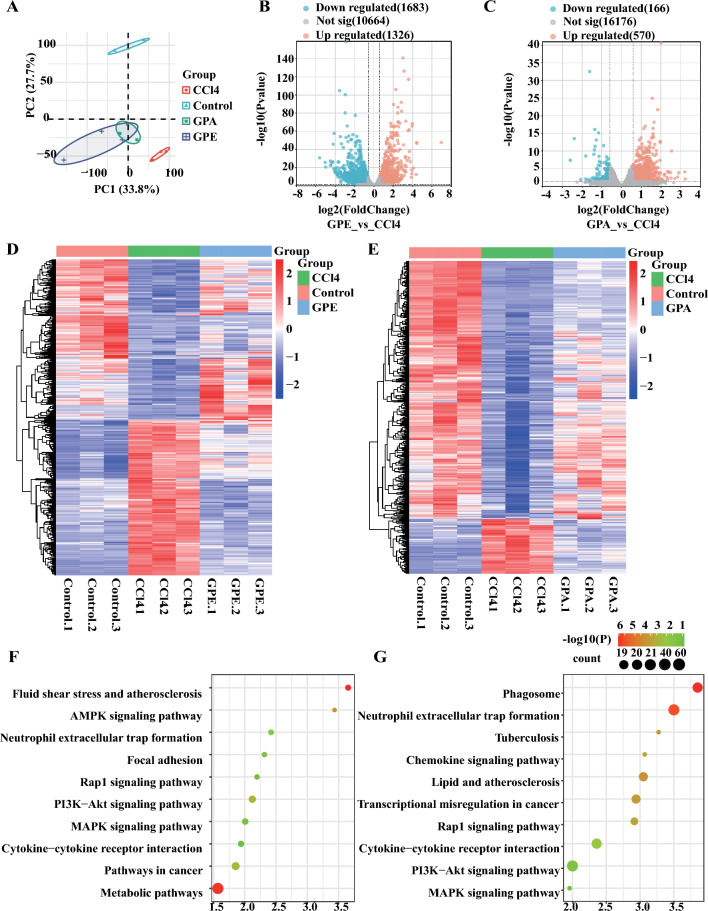
Transcriptomics analysis revealed the signaling pathways involved in the inhibitory effect of GPE and GPA on liver injury. Primary mouse hepatocytes were treated with DMSO, an ethanolic extract of *G. pentaphyllum* (GPE), or Gypenoside A (GPA) in the presence of 8 mM carbon tetrachloride (CCl_4_) for 24 h. mRNA was extracted for transcriptomic analysis. **A** Unsupervised hierarchical clustering and principal component analysis (PCA) diagram (n = 3). **B** Volcano map showing the number of DEGs in the CCl_4_ group versus those in the GPE group (n = 3). **C** Volcano map showing the number of DEGs in the GPA and CCl_4_ group (n = 3). **D**, **E** Heatmap of DEGs in the model group reversed by GPE and GPA administration. Blue indicates downregulated genes, and red indicates upregulated genes (n = 3). **F**, **G** KEGG enrichment analysis of 582 and 482 therapeutic targets of GPE and GPA, respectively

We explored how GPE and GPA reduce apoptosis by performing hepatocyte transcriptomics approach which clarified the potential mechanisms. Unsupervised hierarchical clustering and principal component analysis (PCA) showed that the GPE and GPA groups were close to the control group and far from the CCl_4_ group, suggesting that the gene tended to be normal after the treatment (Fig. 5A). The volcano map showed that compared to the CCl_4_ group, GPE treatment up-regulated 1326 DEGs and down-regulated 1683 DEGs, whereas GPA treatment up-regulated 570 DEGs and down-regulated 116 DEGs (Fig. 5B, C). The Venn diagram showed that GPE treatment reversed the expression of 582 DEGs, whereas GPA treatment reversed the expression of 482 DEGs induced by CCl_4_ (Fig. S3B). These 582 DEGs and 482 DEGs displayed in the heatmaps were considered as the therapeutic targets of GPE and GPA in CCl_4_-induced liver injury (Fig. 5D, E).

KEGG pathway analysis showed that GPE and GPA therapeutic targets of were mainly enriched in PI3K/Akt and MAPK signaling pathways (Fig. 5F). This was consistent with the results of the network pharmacology analysis, which suggested that PI3K/Akt signaling pathway plays a vital role in GPE and GPA in liver injury (Fig. 5F, G). These results implied that GPE and GPA relieved CCl_4_-induced liver injury mainly by inhibiting apoptosis through PI3K/Akt signaling pathway regulation.

### GPE and GPA inhibited liver injury by controlling the PI3K/PDK1/Akt signaling pathway in vivo and in vitro

**Fig. 6:**
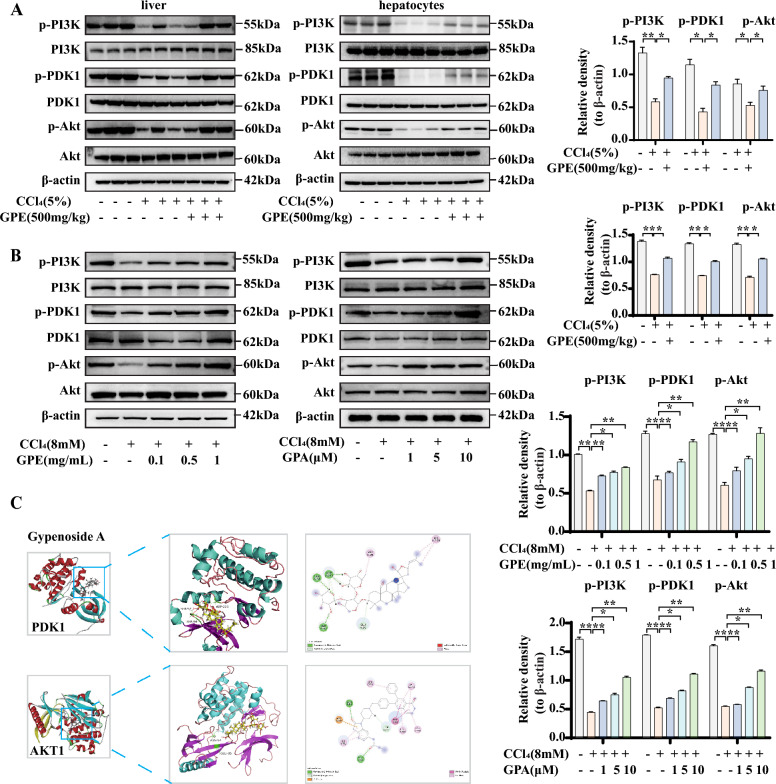
GPE and GPA inhibited liver injury by controlling the PI3K/PDK1/Akt signaling pathway in vivo and in vitro. Mice were intraperitoneally injected with 5% carbon tetrachloride (CCl_4_) or an equivalent amount of olive oil daily for 6 weeks. The mice were injected once daily for the first 2 weeks and then once every 2 d for the next 4 weeks. The ethanol extract of *G. pentaphyllum* (GPE) was administered by gavage at a dose of 500 mg/kg/day from the beginning of modeling for a total of 6 weeks. An equal volume of 0.5% CMC-Na was administered to the control group. **A** Representative western blots for p-PI3K, PI3K, p-PDK1, PDK1, p-Akt, and Akt protein expression in the liver or hepatocytes isolated from the livers of different groups (n = 6). Primary mouse hepatocytes were treated with DMSO, GPE, or GPA in the presence of 8 mM (CCl_4_) for 24 h. **B** Representative western blots showing p-PI3K, PI3K, p-PDK1, PDK1, p-Akt, and Akt protein expression (n = 6). **C** Interaction between the Gypenoside (GPA) and PDK1, as determined by molecular docking. The green dotted line represents the hydrogen bond and interaction between GPA and AKT1 determined by molecular docking. The green dotted line represents the hydrogen bonds. * *p* < 0.05, ** *p* < 0.001: versus indicated group

We measured the protein expression of key molecules in the PI3K/PDK1/Akt in CCl_4_-significantly inhibited induced mice and hepatocytes to confirm whether GPE and GPA treatment affected the pathway. When CCl_4_ significantly inhibited PI3K/PDK1/Akt signaling, GPE treatment restored PI3K/PDK1/Akt signaling by enhancing the phosphorylation of PI3K, PDK1, and Akt (Fig. 6A). Consistent with in vivo, GPE and GPA promoted the expression of p-PI3K, p-PDK1, and p-Akt in the CCl_4_-induced hepatocyte apoptosis model (Fig. 6B). Furthermore, the effect of promoting phosphorylation was dose-dependent. These results demonstrated the inhibitory effects of GPE and GPA on liver injury associated with the PI3K/PDK1/Akt signaling pathway.

We performed a docking analysis to further analyze the direct relationship between GPA and the PI3K/PDK1/Akt signaling pathway. The results of silicon molecular docking showed that GPA had a strong binding ability with Akt and PDK1 (total score: 10.1398 and 4.0415, respectively) but could not bind to PI3K (Fig. 6C). Studies have shown that PDK1 plays an important role in regulating apoptosis via the PI3K/Akt pathway [13]. Therefore, we hypothesized that PDK1 is a potential target protein for GPE and GPA to regulate the PI3K/Akt signaling pathway and improve liver injury.

### PDK1 was required for the effects of GPE and GPA on PI3K/Akt signaling pathway activation and hepatocyte apoptosis

**Fig. 7:**
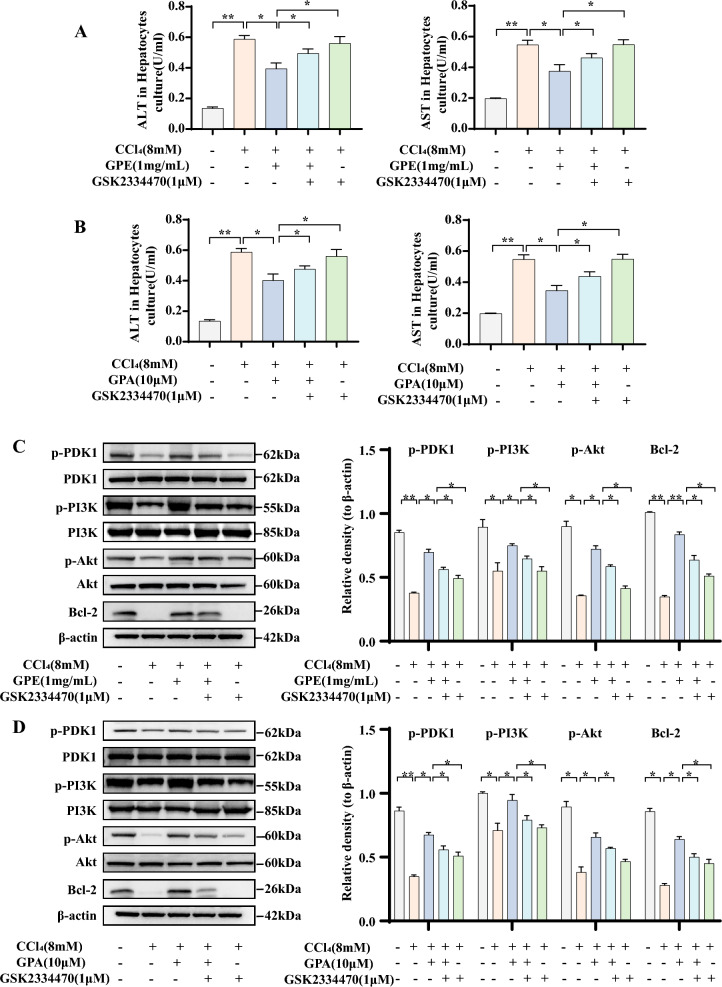
The role of PDK1 inhibition on the modulation of GPE and GPA on hepatocyte apoptosis. Primary mouse hepatocytes were treated with DMSO, the ethanol extract of *G. pentaphyllum* (GPE), or Gypenoside A (GPA), and with or without the PDK1 inhibitor GSK2334470 in the presence of 8 mM carbon tetrachloride (CCl_4_) for 24 h. **A**, **B** Levels of alanine aminotransferase (ALT) and aspartate aminotransferase (AST) in the hepatocyte culture medium (n = 6). **C**, **D** Expression levels of p-PDK1, PDK1, PI3K, p-PI3K, p-Akt, Akt and Bcl-2 in hepatocytes (n = 6). * *p* < 0.05, ** *p* < 0.001: versus indicated group

Silicon docking analysis provided essential clues that GPA mediated PI3K/Akt signaling pathway might interact directly with PDK1. Studies have shown that PDK1 can independently activate the PI3K/Akt signaling pathway. The molecular ablation of PDK1 function is associated with the deactivation of the PI3K/Akt signaling pathway [14]. We used GSK2334470 (1 μM), a novel and highly specific inhibitor of PDK1 [15], to verify the importance of PDK1 to GPE and GPA in CCl_4_-induced hepatocyte apoptosis model. Elevated ALT and AST levels in the cell culture medium showed that GSK2334470 treatment reversed the inhibitory effect of GPE and GPA on cell damage in hepatocytes when exposed to CCl_4_ (Fig. 7A, B). In the present study, GPE and GPA promoted the phosphorylation of PDK1, PI3K, and Akt; however, those effect were abrogated by GSK2334470 treatment (Fig. 7C, D). The results indicated that GSK2334470 inhibited the phosphorylation expression of PDK1, thereby suppressing PI3K and Akt phosphorylation. Concordantly, GSK2334470 treatment attenuated the enhanced effects of GPE and GPA on the anti-apoptotic protein Bcl-2 expression (Fig. 7C, D). These results showed that GPE and GPA enhance the phosphorylation of PDK1, thereby stimulating the PI3K/Akt signaling pathway and subsequently increasing the expression of Bcl-2. This inhibits hepatocyte apoptosis and ameliorates liver injury. GPE and GPA regulated the PI3K/Akt signaling pathway and enhanced the expression of Bcl-2 in a PDK1-dependent manner.

## Discussion

In chronic liver disease, hepatocyte injury can stimulate collagen accumulation and increase matrix stiffness by affecting the metabolic levels in the hepatic microenvironment [16]. Inhibiting hepatocyte apoptosis offers a promising therapeutic approach for liver injury. *G. pentaphyllum*, a perennial plant belonging to the family-Cucurbitaceae, is mainly cultivated in southern China, Japan, and Korea [17]. In this study, we demonstrated that GPE ameliorated liver injury and fibrosis by improving hepatocyte morphology and reducing fibrous accumulation, ultimately inhibiting the liver injury progression.

Liver injury is a pathological consequence of hepatocyte apoptosis. Oxidative stress, toxic injury, inflammation, hypoxia, and viral infections can induce hepatocyte damage, leading to apoptosis [18]. CCl_4_, a hepatorenal toxic agent, effectively simulates the physiological process from hepatocyte damage to apoptosis both in *vivo* and in *vitro*. Therefore, the use of CCl_4_-induced liver injury model and hepatocyte apoptosis models is a promising strategy for drug screening. Our results revealed that GPE reduced the number of TUNEL-positive cells, increased the Bcl-2/Bax ratio, and decreased hepatocyte apoptosis. The discovery of GPE inhibitory effect on hepatocyte apoptosis supplements the insufficient understanding of the GPE mechanism of action in regulating liver disease.

The major bioactive constituents of *G. pentaphyllum* are dammarane-type triterpene saponins known as gypenosides [19]. Approximately 180 gypenosides have been identified in *G. pentaphyllum*, including GPA, GP-XLIX, GP-XLVI, and Rb3. Interestingly, although far from ginseng botanically, *G. pentaphyllum* also contains ginsenosides including Rb3. The ginsenosides in *G. pentaphyllum* constitute approximately 25% of the total saponins in the plant. *G. pentaphyllum* is an essential and unique plant outside the family-Araliaceae (i.e., ginseng, notoginseng, and American ginseng) that contains ginsenosides. Therefore, *G. pentaphyllum* has been praised as “Southern ginseng” and “poor man’s ginseng” [17], which can be explained by the chemical resemblance of *G. pentaphyllum* saponins to those of Araliaceae family *Panax* species. Studies have shown that *G. pentaphyllum* has some of the benefits similar to those of ginseng. In this study, we found that GPA could prevent CCl_4_-induced hepatocyte apoptosis in vitro, GP-XLIX and GP-XLVI had no significant influence on the anti-apoptotic phenotype, and although Rb3 tended to alleviate apoptosis, its effects were weaker than those of GPA. Therefore, we believe that GPA is the primary active substance in *G. pentaphyllum* that exerts anti-apoptotic activity, which explains the distinction between the hepatoprotective effects of *G. pentaphyllum* and ginseng.

We conducted a transcriptomic analysis using bioinformatics to elucidate further the specific mechanism by which GPE and GPA exert anti-apoptotic effects on hepatocytes. KEGG enrichment analysis revealed that the anti-apoptotic mechanisms of GPE and GPA may be associated with the PI3K/Akt, MAPK, and Rap1 signaling pathways. Akt activation promotes phosphorylation of the Bcl-2/Bcl-XL-related death promoter (Bad), thereby inhibiting caspase-3-mediated cell death [20]. This phosphorylation event leads to the dissociation of Bcl-2 from phosphorylated Bad and promotes cell survival [21]. Research has demonstrated that PI3K/Akt pathway activation can elevate Bcl-2 protein levels, decrease Bax and caspase 3 proteins expression, confer protection against apoptosis, and ameliorate liver diseases [22, 23]. Given that our previous results confirmed that GPE can elevate the decrease in the Bcl-2/Bax ratio induced by CCl_4_ and considering its relationship with the PI3K/Akt pathway, we hypothesized that GPE and GPA might exert their anti-apoptotic effects through the PI3K/Akt signaling pathway. As expected, we found that GPE and GPA increased the protein levels of p-PI3K and p-Akt in the PI3K/Akt pathway, thereby enhancing the Bcl-2/Bax ratio.

PDK1 is a phosphorylation-regulated kinase expressed in various eukaryotic organs that plays a central role in activating diverse cell signaling pathways [24, 25]. PI3K activation generates phosphatidylinositol-3,4,5-triphosphate [26], and subsequently recruits PDK1 and Akt to the plasma membrane. Membrane-bound PDK1 then phosphorylates Akt at Thr308, thereby activating Akt and initiating the PI3K/Akt pathway, ultimately suppressing apoptosis and autophagy [27]. According to previous research, PDK1 serves as an independent activator of the PI3K/Akt signaling pathway, and silencing the expression of PDK1 affects the expression of the PI3K/Akt signaling pathway [14]. Consequently, we further investigated the critical role of PDK1 in GPE inhibition of cell apoptosis and improvement of liver injury. GPE treatment restored PI3K/PDK1/Akt signaling, which was inhibited by CCl_4_ by enhancing the phosphorylation of PI3K, PDK1, and Akt in vivo. GPE and GPA promoted the expression of p-PI3K, p-PDK1, and p-Akt in a CCl_4_-induced hepatocyte apoptosis model. Docking analysis showed that GPA had a strong binding ability to Akt and PDK1, but could not bind to PI3K. In the CCl_4_-induced hepatocyte apoptosis model, the addition of GSK2334470, a novel and highly specific PDK1 inhibitor, weakened the effects of GPE and GPA on hepatocyte apoptosis. This was achieved by deactivating PDK1 phosphorylation and inhibiting the phosphorylation of PI3K and Akt. These findings suggest that GPE and GPA enhance the activity of PDK1 by regulating its phosphorylation, which in turn activates the PI3K/Akt signaling pathway. These results indicated that GPE and GPA regulated the PI3K/Akt signaling pathway and enhanced the expression of Bcl-2 in a PDK1-dependent manner.

**Fig. 8:**
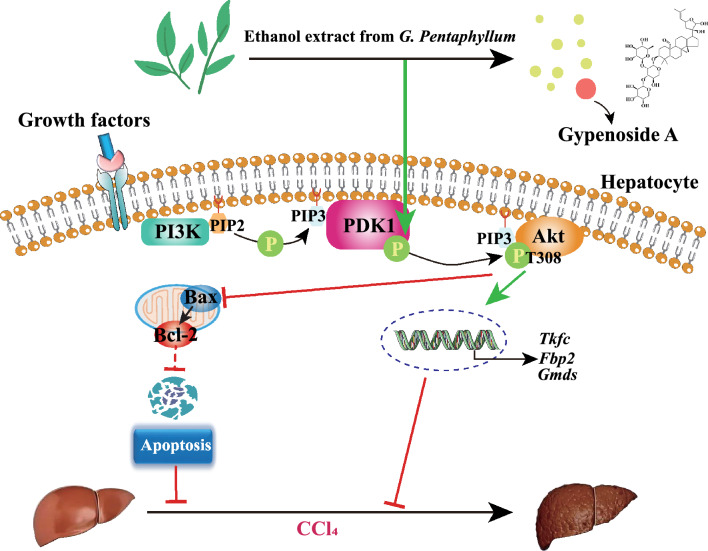
The ethanol extract of *G. pentaphyllum* (GPE) and its unique saponin Gypenoside A (GPA) significantly ameliorates CCl_4_-induced liver injury and fibrosis via inhibiting hepatocyte apoptosis activation by controlling PDK1-mediated PI3K/Akt signaling pathway to regulate the Bcl-2 and caspase families expression

In conclusion, *G. pentaphyllum* exerts significant therapeutic effects against CCl_4_-induced liver injury and fibrosis by inhibiting hepatocyte apoptosis and controlling the PDK1-mediated PI3K/Akt signaling pathway to regulate Bcl-2 and caspase family expression (Fig. 8). Our findings contribute to a comprehensive understanding of the protective effects of GPE and GPA against liver injury and may provide a potential remedy for treating liver diseases in the future.

### Supplementary Information

Supplementary material 1.

## Data Availability

The data that support the findings of this study are available from the corresponding author upon reasonable request.

## Abbreviations

AST: Aspartate aminotransferase
ALT: Alanine aminotransferase
Akt: Protein kinase B
CCl_4_: Carbon tetrachloride
COL1A1: Collagen 1A1
DEGs: Differentially expressed genes
Ginseng: *Panax ginseng* (C. A. MEY)
GPA: Gypenoside A
GPE: Ethanol extract from *Gynostemma pentaphyllum* (thunb.) makino
GP-XLIX: Gypenoside XLIX
GP-XLVI: Gypenoside XLVI
H&E: Haematoxylin and eosin
HYP: Hydroxyproline
HSCs: Hepatic stellate cells
KEGG: Kyoto Encyclopedia of Genes And Genomes
PCA: Principal component analysis
PDK1: Phosphoinositide dependent protein kinase-1
PI3K: Phosphatidylinositol-3-kinase
PPI: Protein–protein interaction
Rb3: Ginsenoside Rb3
α-SMA: Alpha smooth muscle
TCM: Traditional Chinese medicine
TUNEL: Terminal Deoxynucleotidyl Transferase mediated dUTP Nick-End Labeling
