# Supplementary material for: Gynostemma Pentaphyllum ameliorates CCl4-induced liver injury via PDK1/Bcl-2 pathway with comprehensive analysis of network pharmacology and transcriptomics
Source: Chin Med. 2024 May 15;19:70. doi: 10.1186/s13020-024-00942-w (PMC11094861; doi:10.1186/s13020-024-00942-w)
Supplement: Supplementary file 1 — Supplementary material 1. [file 13020_2024_942_MOESM1_ESM.pdf]

SUPPLEMENTARY MATERIALS

Supplementary Table 1. The antibody information.

Name	Supplier	Cat no.	Gene ID
α-SMA	CST	19245S	59
COL1A1	CST	72026S	1277
Bcl-2	ABclonal	A20777	596
Bax	ABclonal	A19684	581
cleaved caspase-3	ABclonal	A11040	836
cleaved caspase-7	Affinity	AF4023	840
cleaved-PARP	ABclonal	A22535	11545
PI3K	ABclonal	AP0584	5295
p-PI3K	ABclonal	A4992	5295
PDK1	ABclonal	A1665	5170
p-PDK1	ABclonal	AP0477	5170
Akt	CST	9272S	207
p-Akt	CST	13038S	207
β-actin	ABclonal	AC006	60

Supplementary Table 2. The compounds of *G. pentaphyllum* information.

Mol ID	Molecule Name	MW	OB (%)	DL
MOL000338	3'-methyleriodictyol	302.3	51.61	0.27
MOL000351	Rhamnazin	330.31	47.14	0.34
MOL000359	sitosterol	414.79	36.91	0.75
MOL004350	Ruvoside_qt	390.57	36.12	0.76
MOL004355	Spinasterol	412.77	42.98	0.76
MOL005438	Campesterol	400.76	37.58	0.71
MOL005440	Isofucosterol	412.77	43.78	0.76
MOL000953	CLR	386.73	37.87	0.68
MOL000098	quercetin	302.25	46.43	0.28
MOL009855	(24S)-Ethylcholesta-5,22,25-trans-3beta-ol	410.75	46.91	0.76
MOL009877	cucurbita-5,24-dienol	426.8	44.02	0.74
MOL009888	Gypenoside XXXVI	458.8	37.85	0.78
MOL009969	Gypenoside XXXVI	444.77	37.73	0.78
MOL009971	Gypenoside XXVII	418.73	30.21	0.74
MOL009973	Gypenoside XXVIII	416.71	32.08	0.74
MOL009986	Gypentonoside A	472.78	36.13	0.8

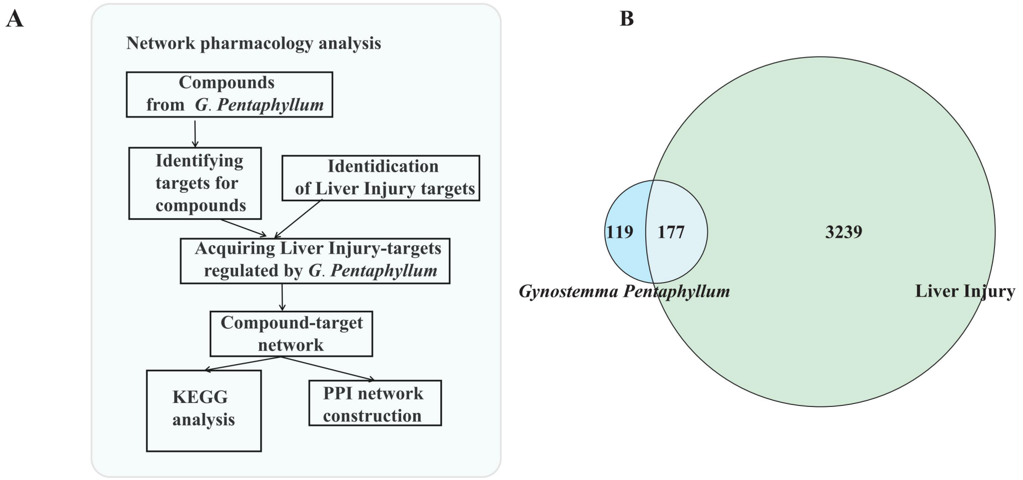

Fig. S1. Network pharmacology predicted potential signaling pathways in the inhibitory effect of GPE on liver injury.

Network pharmacology analysis was applied to predict the targets of GPE on liver fibrosis. (A) The protocol of network pharmacology analysis in this study. (B) Venny diagram of overlapping genes from GPE and liver fibrosis.

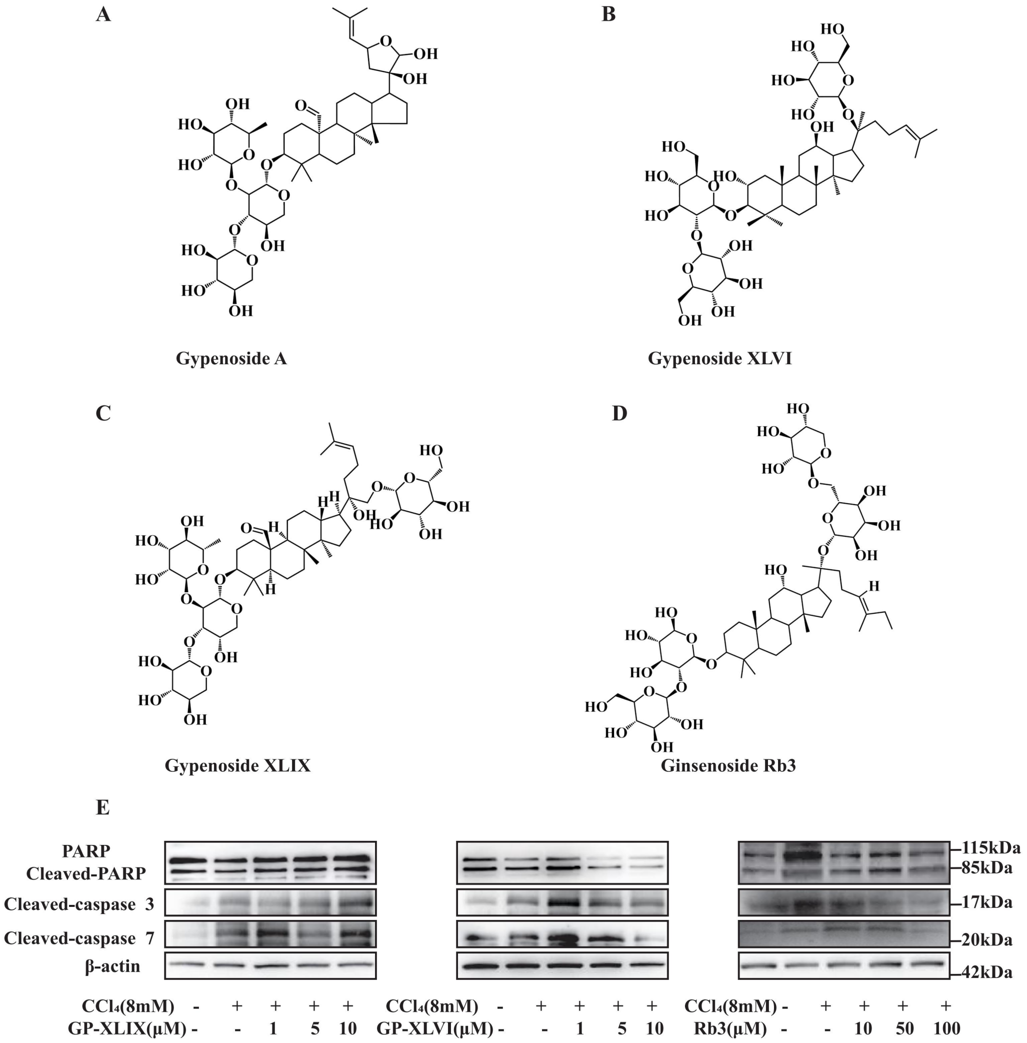

Fig. S2. The anti-apoptotic effects of the main triterpenoid isolated from GPE on the CCl_4-induced hepatocyte apoptosis model.

Primary mouse hepatocytes were treated with DMSO or *Ginsenoside* Rb3 (Rb3) or *Gypenoside* XLIX (GP-XLIX) or *Gypenoside* XLVI (GP-XLVI) in the presence of 8mM carbon tetrachloride (CCl_4) for 24 h. (A, B, C, D) Chemical structures of four gypenosides. (E) The protein expression levels of PARP/Cleaved-PARP, Cleaved-caspase 3 and Cleaved-caspase 7 in the hepatocytes.

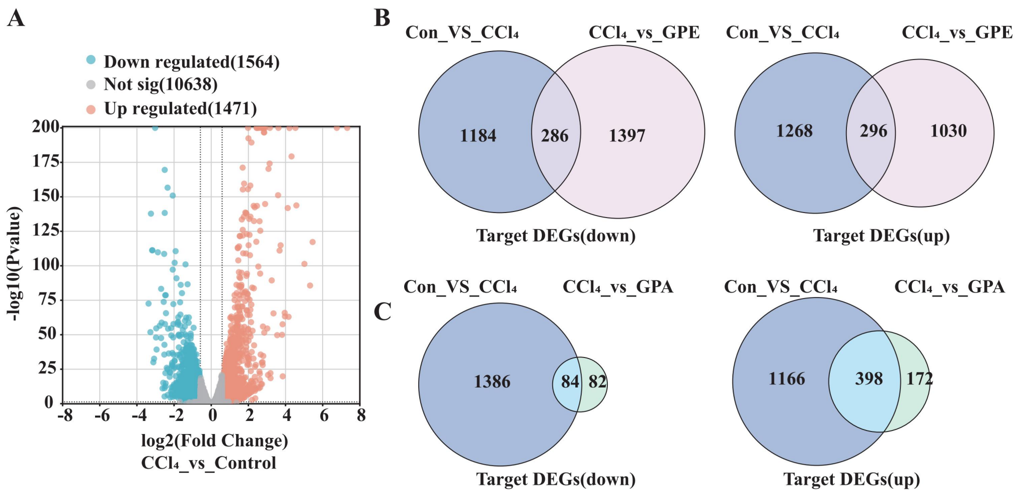

Fig. S3. Transcriptomics analysis revealed the signaling pathways involved in the inhibitory effect of GPE and GPA on liver injury.

Primary mouse hepatocytes were treated with DMSO, GPE or GPA in the presence of 8mM carbon tetrachloride (CCl_4) for 24 h. Then, the mRNA was extracted for transcriptomic analysis. (A) Volcano map showed the number of DEGs in CCl_4 group versus Control group (n=3). (B) Venn diagrams showed the number of DEGs that were upregulated in CCl_4 group (CCl_4 *vs.* Control, Upregulated DEGs) and downregulated by GPE (GPE *vs*. CCl_4 , Downregulated DEGs), and DEGs that were downregulated in CCl_4 group (CCl_4 *vs.* Control, Downregulated DEGs) and upregulated by GPE (GPE *vs*. CCl_4, Upregulated DEGs) (n=3). (C) Venn diagrams showed the number of DEGs that were upregulated in CCl_4 group (CCl_4 *vs.* Control, Upregulated DEGs) and downregulated by GPA (GPA *vs*. CCl_4 , Downregulated DEGs), and DEGs that were downregulated in CCl_4 group (CCl_4 *vs.* Control, Downregulated DEGs) and upregulated by GPA (GPA *vs*. CCl_4, Upregulated DEGs) (n=3)
